# Accumulation of Fascin^+^ cells during experimental autoimmune neuritis

**DOI:** 10.1186/1746-1596-8-213

**Published:** 2013-12-25

**Authors:** Zichen Yang, Jian Sun, Xiaofeng Yang, Zhiyuan Zhang, Bangwei Lou, Jian Xiong, Hermann J Schluesener, Zhiren Zhang

**Affiliations:** 1 Institute of Immunology, Third Military Medical University of PLA, 30 Gaotanyan Main Street, Chongqing 400038, People’s Republic of China; 2 Division of Immunopathology of the Nervous System, University of Tuebingen, Calwer Street 3, Tuebingen D-72076, Germany

**Keywords:** EAN, Fascin, Dendritic cells

## Abstract

**Background:**

Experimental autoimmune neuritis (EAN) is a well-known animal model of human demyelinating polyneuropathies and is characterized by inflammation and demyelination in the peripheral nervous system. Fascin is an evolutionarily highly conserved cytoskeletal protein of 55 kDa containing two actin binding domains that cross-link filamentous actin to hexagonal bundles.

**Methods:**

Here we have studied by immunohistochemistry the spatiotemporal accumulation of Fascin + cells in sciatic nerves of EAN rats.

**Results:**

A robust accumulation of Fascin + cell was observed in the peripheral nervous system of EAN which was correlated with the severity of neurological signs in EAN.

**Conclusion:**

Our results suggest a pathological role of Fascin in EAN.

**Virtual slides:**

The virtual slides for this article can be found here: http://www.diagnosticphatology.diagnomx.eu/vs/6734593451114811

## Introduction

Experimental autoimmune neuritis (EAN) is an autoantigen-specific T-cell mediated inflammatory demyelinating disease of the peripheral nervous system (PNS), which is characterized by weight loss, ascending paraparesis/paralysis and spontaneous recovery [1]. The EAN model shares many clinical, electrophysiological and immunological features with the human acute and chronic inflammatory demyelinating polyradiculoneuropathies (AIDP and CIDP) and is therefore widely applied to investigate the disease mechanisms and therapeutic principles of AIDP and CIDP. EAN can be actively induced with peripheral nerve autoantigen and is pathologically characterized by breakdown of the blood-nerve barrier (BNB), robust accumulation of reactive T cells and macrophages into the PNS and demyelination of peripheral nerves [2].

In EAN, T cells are activated by autoantigen following immunization, and then attached to the venular endothelium in the PNS and penetrate the BNB. The infiltrated T cells amplify the local inflammation by recruiting more T cells and macrophages via chemokines and cytokines [3-5]. Subsequently, the breakdown of the BNB allows the passage of the circulating autoantibodies that synergize with T cells and macrophages to cause demyelination [2]. EAN has been considered to be mainly mediated by Th1 helper cells and Th1 cytokines [6]. Th1 cells are activated by antigen-presenting cells (APCs) that are key players during both the initiation and progression of the autoimmune response. Dendritic cells (DC) are specialized APC that most efficiently present antigen to naive T cells and thus initiate the primary immune response [7].

Fascin is an evolutionarily highly conserved cytoskeletal protein of 55 kDa containing two actin binding domains that cross-link filamentous actin to hexagonal bundles [8]. Fascin is involved in cell motility, as has been shown by intracellular treatment with inhibitory anti-Fascin antibody [9] and by Fascin overexpression [10]. Fascin is distributed in mature DCs [11], especially including the numerous filopodia-like dendritic cell extensions [12]. Moreover, Fascin expression is now becoming adopted as a reliable maturation maker for DCs [8], which may facilitate identification and quantification of mature DCs in tissue samples or cultured differentiated populations [13]. For example, Fascin is applied as a marker for mature DCs in anticancer therapies that target DCs, where isolation of pure DCs is a crucial requirement [14], while loss of Fascin-positive DCs in follicular lymphomas has been suggested to contribute to the clinical prognosis [15]. Functionally, Fascin is important for the migration of activated DCs [16]. Moreover, Fascin-dependent dendrites of DCs might be involved in the formation and maintenance of contact to T cells [17]. Because filamentous actin and Fascin were found to be focally polarized in DCs at the immunologic synapse formed with clustered allogeneic Th cells. Furthermore, T cell proliferation was markedly decreased after pretreatment of murine bone marrow-derived DCs with Fascin-directed antisense oligonucleotides.

While accumulated data have shown DCs to be key players in autoimmunity the distribution of DCs in PNS of EAN still remains unknown. So here we analyzed the spatiotemporal expression of Fascin in sciatic nerves of EAN rats.

## Materials and methods

### Animal experiments and tissue library

Male Lewis rats (8–10 weeks old, 200–250 g, Charles River, Sulzfeld, Germany) were housed with equal daily periods of light and darkness, and free access to food and water. All procedures were performed in accordance with the published European Health Guidelines under a protocol approved by the local Administration District Official Committee, and all efforts were made to minimize the number of animals and their suffering. EAN was induced by s.c. Immunization with an emulsion of 100 μg of synthetic neuritogenic P2 peptide/CFA as described [18]. The severity of EAN was scored daily as follows: 0-normal, 1-reduced tonus of tail, 2-limp tail, impaired righting, 3-absent righting, 4-gait ataxia, 5-mild paresis of the hind limbs, 6-moderate paraparesis, 7-severe paraparesis or paraplegia of the hind limbs, 8-tetraparesis, 9-moribund, 10-death. In our present study, a previously reported EAN sciatic nerve library [18], containing tissue at days 7, 11, 13, 15, 17 and 22 (three rats each time point) was used. In addition, tissue from five normal rats was used as normal control.

### Tissue preparation and immunohistochemistry (IHC)

Rats were deeply anesthetized with ether and perfused intracardially with 4°C, 4% paraformaldehyde (PFA in PBS). Sciatic nerves were quickly removed and post-fixed in 4% PFA overnight at 4°C. Sciatic nerves were cut into two equally long segments. All of these segments were embedded in paraffin, sectioned serially (3 μm) and mounted on silane-covered slides. IHC was performed on 3 μm paraffin-embedded sections using purified polyclonal antibody against Fascin, which is considered DC marker. The specificity of this antibody has been demonstrated by ELISA and Western blotting (product datasheet, Santa Cruz Biotechnology). The specificity of these antibodies has been demonstrated by ELISA and Western blotting (product data sheets, Sigma and Serotec). After dewaxing, sections were boiled (in an 850 W microwave oven) for 15 min in citrate buffer (2.1 g citric acid monohydrate/l, pH 6) (Carl Roth, Karlsruhe, Germany). Endogenous peroxidase was inhibited by 1% H2O2 in pure methanol (Merck, Darm-stadt, Germany) for 15 min. Sections were incubated with 10% normal pig serum (Biochrom, Berlin, Germany) to block non-specific binding of immunoglobulins and then with Fascin antibodies overnight at 4°C. Antibody binding to tissue sections was visualized with biotinylated swine anti-rabbit IgG antibody (1:400; Dako, Hamburg, Germany) and subsequent incubation with a horseradish peroxidase-conjugated streptavidin complex for 30 min (1:100; Dako), followed by development with diaminobenzidine (DAB) substrate (Fluka, Neu-Ulm, Germany). Finally, sections were counterstained with hemalum. As negative controls for immunostaining, the primary antibodies were omitted. By control staining, no immunoreactivity (IR) was detected in EAN sciatic nerves without primary Fascin antibody.

### Double staining

In double-staining experiments, sciatic nerve sections were immunolabeled as described above. Then, they were once more irradiated in a microwave for 15 minutes in citrate buffer and were incubated with 10% normal pig serum (Biochrom). Subsequently, the sections were incubated with the appropriate second primary monoclonal and polyclonal antibodies for 1 h at room temperature. The following antibodies were used: ED1 (1:100; Serotec, Oxford, UK) for activated microglia/macrophages, CD3 (1:50; Serotec) for T-lymphocytes. Consecutively, visualization was achieved by adding secondary antibody at a dilution of 1:400 in tris buffered saline with bovine serum albumin (TBS-BSA) for 30 minutes, and then alkaline phosphatase-conjugated Avidin complex diluted 1:100 in TBS-BSA for another 30 minutes. Finally, immunostaining was developed with Fast Blue BB salt chromogen-substrate solution, but by omission of counterstaining with hemalum.

### Data acquisition and statistical analysis

After immunostaining, sections of each time point were examined by light microscopy for expression of Fascin. To evaluate immunostaining data, the numbers of positively stained cells to areas of sciatic nerve cross-sections were calculated. Briefly, images of sciatic nerve cross-sections were captured under 40 × magnification using Nikon Cool-scope (Nikon, Duesseldorf, Germany) with fixed parameters. Images were analyzed using Metamorph Off-line 7.1 (Molecular Devices, Toronto, Canada). Positively stained cells were counted by two independent observers from randomly chosen areas. For each EAN rat, four cross-sections from root and middle levels of both sides were analyzed. Results were given as arithmetic means of numbers of positively stained cells to areas of sciatic nerve cross-sections and standard errors of means (SEM). Differences in numbers of positively stained cells per unit of area of cross-sections among different time points were analyzed by one-way ANOVA followed by Dunnett’s multiple comparison test (GraphPad 5.0 for Windows, GraphPad Software, San Diego, USA), and data presented as histograms created by GraphPad Prism 5.0 for Windows. Relative abundance of cell populations was presented as the ratio of arithmetic means. Correlation analysis was evaluated by checking Spearman’s correlation coefficient (for nonparametric correlation) (GraphPad Prism 5.0 for windows). For all statistical analyses, significance levels were set at P < 0.05.

## Results

### Fascin expression in rat sciatic nerves following EAN

Rat EAN model has been established and the time course of neurological scores of our EAN library has been published previously [18]. For this EAN tissue library, the first neurological sign (reduced tonus of tail) was seen at around day 12 (1.3 ± 0.8) and the maximal severity of neurological signs appeared at about 15 days after immunization (5.3 ± 0.3). Thereafter, rats rapidly recovered from EAN and no neurological signs were observed by day 22.

**Figure 1:**
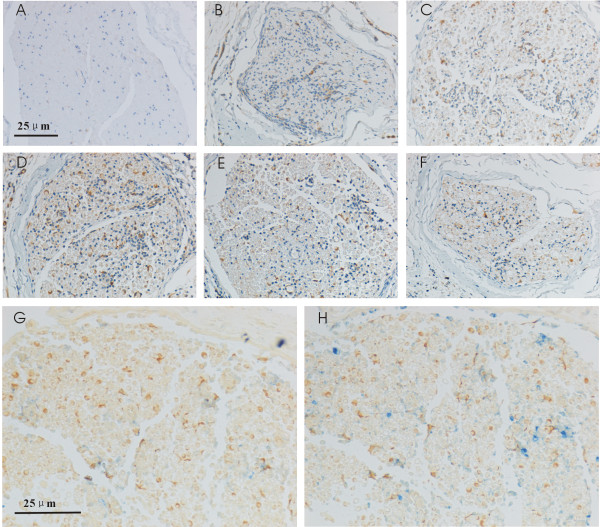
**Immunohistochemical staining of Fascin in sciatic nerves of EAN rats.** In normal rat sciatic nerves, Fascin expression was only occasionally observed in single cells **(A)**. At day 7, the Fascin^+^ cells were observed clearly **(B)**. Significant accumulation of Fascin^+^ cells could be observed at day 11 **(C)**, maximal accumulation of Fascin^+^ cells in sciatic nerves was found at day 15 **(D)**. From day 17, the accumulation of Fascin^+^ cells began to decrease **(E)**, but finally the cellular accumulation returned to a control level by day 22 **(F)**. Double staining experiments revealed that few Fascin^+^ cells co-expressed cd-3 (blue) representing T cells **(G)**, and few Fascin^+^ cells co-expressed active macrophage marker ED1 (blue)at day 15 **(H)**. Scale bars are 25 mm for all pictures.

Fascin expression in rat sciatic nerves of EAN rats were studied by IHC. Results are given as means of numbers of Fascin^+^ cell ± ESM per mm^2^, and compared to day 0 (Figure 1A). Following immunization, slight accumulation of Fascin^+^ cells was observed already at Day 7 (Figure 1B) and increases dramatically to Day 11 (Figure 1C). The maximum accumulation of Fascin^+^ cells was observed at Day 15 (Figure 1D), corresponding to the peak of disease severity. Subsequently, a considerable decrease of Fascin^+^ cells was observed and finally returned to normal control level by Day 22 (Figure 1E and F).

### Correlation of Fascin^+^ cell accumulation with EAN severity

**Figure 2:**
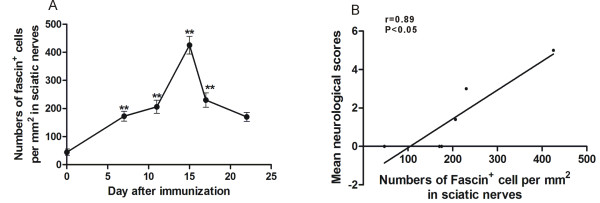
**Fasin**^**+ **^**cell accumulations in sciatic nerves and correlation with severity of neurological signs. A**: The time course of lesional Fascin^+^ cell numbers in sciatic nerves. **B**: A significant positive correlation between the time course of Fascin^+^ cells accumulation in sciatic nerves and the time course of neurological scores of EAN rats was observed. *: p < 0.05 and **: p < 0.01 compared to Day 0.

In sciatic nerves, the accumulation of Fascin^+^ cells appeared earlier compared with the development of neurological signs. However, the maximum levels of Fasin^+^ cells and clinical scores were found at the same day (day 15), and the significant accumulation of Fascin^+^ cells were observed in accordance with the severity of EAN. Further correlation analysis proved a significant positive correlation of the time course of Fascin^+^ cells accumulation in sciatic nerves with the time course of neurological scores of EAN rats in our observation ( r =0.89; P < 0.05) (Figure 2).

### Double-labeling experiments

We further characterized the accumulated Fascin^+^ cells by double labeling with monoclonal antibodies directed against activated macrophages (ED1) and T cells (CD3). A double-staining experiment was performed in sciatic nerves of Day 15 EAN rats. Few co-expression of Fascin and CD3 was observed at day 15 (Figure 1G) and few Fascin^+^ cells co-expressed active macrophage marker ED1 (blue) at day 15 (Figure 1H). As DC is the only one that has Fascin in the white blood cells, so we think the infiltrating Fascin^+^ cells around vessels are DCs.

## Discussion

In our study, the accumulation of Fascin^+^ cells in sciatic nerves of EAN rats was investigated for the first time. The maximal Fascin^+^ cells number was recorded at day 15, which is in accordance with the severity of EAN. A significant positive correlation of Fascin^+^ cell accumulations with the time course of neurological scores has been demonstrated. Moreover, Fascin^+^ cells were not uniformly distributed, but were mainly observed concentrated around vessels and the major Fascin^+^ cells were dendritic cells in sciatic nerves.

Fascin is a 55 kDa, actin-binding protein that regulates the rearrangement of cytoskeletal elements, and interactions between the cytoskeleton and the cell membrane [9]. Among leukocytes, Fascin expression has been demonstrated only in mature DCs [15]. Our study also found that the major Fascin^+^ cells were dendritic cells in sciatic nerves, which has also been reported by Hobbenaghi *et al.*[19]. Moreover, the expression of Fascin in DCs might be associated with dendrite formation for it has been proved necessary for the formation of the dendritic processes of maturing Langerhans cells [20]. Thus, Fascin is suggested to have a role in the T and DCs interaction [17] and the initiation of consequent adaptive immune response.

We observed an accumulation of Fascin inflammatory cells in sciatic nerves of EAN rats. The up-regulation of Fascin has been reported in several other autoimmune diseases, like Multiple Sclerosis [21], systemic lupus erythematous (SLE) [22] and autoimmune cerebellar ataxia [23]. Particularly in EAE, the analog of EAN in the CNS, a robust expression of Fascin was observed, and the expression was also associated with the time course of EAE [24]. In our study, upregulation of Fascin was observed before the onset of EAN and reached the maximal level shortly before the peak of neurological severity. The correlation indicates a Fascin involvement of adaptive immunity in the effector phase of EAN.

In DCs, the actin cytoskeleton is essential for the formation of its characteristic dendrite, as well as an immunological synapse [25]. It has been previously reported as an active role of formation of the immunological synapse in mature DCs [7]. DCs reorganize their actin cytoskeleton and show polarized exocytosis of cytokines towards bound T cells. Disruption of the actin cytoskeleton in DCs severely diminishes T cell activation [7]. Since T cells are key players in the development of EAN, the correlative Fascin accumulation in DCs might offer interests in further comprehension of the mechanism of EAN and relative autoimmune diseases.

## Conclusions

In summary, here we observed a significant upregulation of Fascin in EAN sciatic nerves and that is correlated with disease severity. Our results suggest that Fascin based immuno-modulation may play an important pathological role in the EAN and warrants further investigation.

## Competing interests

The authors declare that they have no competing interests.

## Authors’ contribution

ZRZ pervised the conduction of the whole project, and designed ZCY contributed to the establishment of EAN model in rats, performed the experiments and drafted the manuscript; JS and XFY analysed data and did part of the animal study, sample collection and measurement; ZYZ performed the double staining experiments in German; BWL and JX contributed to revise the manuscript; HJS offered great advises during the whole process of this project; All authors read and approved the final manuscript.
